# Assessing bleeding risk in 4824 Asian patients with atrial fibrillation: The Beijing PLA Hospital Atrial Fibrillation Project

**DOI:** 10.1038/srep31755

**Published:** 2016-08-25

**Authors:** Yu-tao Guo, Ye Zhang, Xiang-min Shi, Zhao-liang Shan, Chun-jiang Wang, Yu-tang Wang, Yun-dai Chen, Gregory Y. H. Lip

**Affiliations:** 1 Department of Cardiology, Chinese PLA General Hospital, Beijing, China; 2 Health Division of Guard Bureau, Chinese PLA General Staff Department, Beijing, China; 3 University of Birmingham Centre for Cardiovascular Sciences, City Hospital, Birmingham, United Kingdom

## Abstract

The risks of major bleeding and intracranial hemorrhage (ICH) are higher in Asian patients with atrial fibrillation (AF) compared to non-Asians. We aimed to investigate risk factors for bleeding, and validate the predictive value of available bleeding risk scores (mOBRI, HEMORR_2_HAGES, Shireman, HAS-BLED, ATRIA and ORBIT) in a large cohort of Chinese inpatients with AF. Using hospital electronic medical databases, we identified 4824 AF patients (mean age 67 years; 34.9% female) from January 1, 1995 to May 30, 2015, with median (interquartile) in-hospital days of 10 (7–16) days. On multivariate analysis, prior bleeds, vascular disease, anemia, prior stroke, and liver dysfunction were independent risk factors of major bleeding (all p < 0.05). C-statistics (95%CI) of the HAS-BLED score were 0.72 (0.65–0.79) for major bleeding events and 0.83 (0.75–0.91) for ICH (all p < 0.001). Compared to other risk scores, the HAS-BLED score was significantly better in predicting major bleeding events (Delong test, all P < 0.05, apart from mOBRI, HEMORR_2_HAGES) and ICH (all p < 0.05), and additionally, resulted in a net reclassification improvement (NRI) of 17.1–65.5% in predicting major bleeding events and 29.5–67.3% in predicting ICH (all p < 0.05). We conclude that the HAS-BLED score had the best predictive and discriminatory ability for major bleeding and ICH in an Asian/Chinese AF population.

Atrial fibrillation (AF) is a global health burden, which may be more serious in developing countries^1^, given that the prevalence of AF and AF-related stroke has significantly increased during recent ten years in countries such as China^2^,^3^. Compared to Western populations, the Asian population with AF may have different clinical profile related stroke risk and thromboprophylaxis^4^. For example, Asian patients are especially prone to intracranial haemorrhage (ICH), and developing countries bear 80% of the global burden of ICH^5^. The higher risk of ICH with Asian subjects, in comparison to the white Caucasian population, lies in not only amongst patients taking warfarin, but also in non-anticoagulated subjects^6^,^7^. AF patients in Asia seem to be at higher risk of ICH, even with a lower rate of anticoagulant use and lower target of International Normalized Ratio (INR). Suboptimal thromboprophylaxis is common in the Asian population with AF^8^, partly due to concerns about ICH and perhaps, the lack of a simple easy way to assess bleeding risk in Asians.

Most studies on bleeding risk (and bleeding risk stratification schemes) in patients AF have been based on predominantly white populations taking oral anticoagulant (OAC) in North America or Europe^9^. Some factors associated with bleeding events have been recognized, but the possibility of varies between Asia and the rest of world in relation to various risk factors and co-morbidities associated with AF^10^.

Several bleeding risk scores have been proposed, such as the modified Outpatient Bleeding Risk Index (mOBRI) in 1989^11^, HEMORR_2_HAGES (Hepatic or renal disease, Ethanol abuse, Malignancy, Older age, Reduced platelet count or function, Rebleeding risk, Hypertension, Anemia, Genetic factors, Excessive fall risk, Stroke) score in 2006^12^, the Shireman score in 2006^13^, HAS-BLED (hypertension, abnormal renal/liver function, stroke, bleeding history or predisposition, labile INR, elderly, drugs/alcohol concomitantly) in 2010^14^, ATRIA (AnTicoagulation and Risk factors In Atrial fibrillation) in 2011^15^ and most recently, the ORBIT (national Outcomes Registry for Better Informed Treatment of Atrial Fibrillation) score^16^.

The HAS-BLED score has been validated in several population cohorts (including untreated, as well as aspirin, warfarin and non-warfarin anticoagulant users) and is the only score predictive of ICH^17^,^18^,^19^,^20^,^21^,^22^,^23^. However, there are limited data in Asian/Chinese cohorts^17^. The predictive value of the various bleeding risk scores and their relative predictive performance to each other for major bleeding and ICH, is uncertain among the Asian population.

The present study aims to investigate the risk factors contributing to major bleeding and second, to validate the predictive value of available risk scores for major bleeding and ICH risk in a large cohort of Chinese patients with AF.

## Methods

The Chinese PLA General Hospital is a comprehensive medical and teaching institution in Beijing, which has 125 clinical, medical and technological departments, 4000 patient beds, and in 2014 had annual patient activity of more than 3.8 million outpatient visits, over 110000 admissions and more than 65000 operations year. The hospital has provided comprehensive health and medical care to civilian patients and troops in the Beijing area, as well as the critically-ill who are transferred from different areas of China outwith Beijing. Attending patients have a permanent and personal registration number in the hospital, which allows accurate and complete collection of patient’s hospital visits. Every medical “event” could be identified through the patient’s permanent and personal registration number.

For this study we used the PLA General Hospital electronic medical records database which recorded the patient’s medical history, therapeutic procedure, mortality data, laboratory data (based on Laboratory Information System, LIS), and imaging data (based on Picture Archiving and Communications System, PACS). The patients were identified with a primary diagnosis of AF or with a major co-morbid diagnosis (i.e. Secondary diagnosis) of AF (International Classification of Disease, Ninth Revision [ICD-9]/International Classification of Disease, tenth Revision [ICD-10] codes 427.3, 427.31/I48) between January 1, 1995 to May 30, 2015. ICD-10 codes have been used since 2008 in the PLA General Hospital, and their accuracy has been validated in prior studies^17^.

The study was carried out in accordance with the principles and rules of Medical Ethics Committee of PLA General Hospital, which has been approved by China Food and Drug Administration (CFDA) (Registry number: XZF20120145), and the medical ethics committee of PLA General Hospital approved the present study (Approval No. S2013-064-02). There was no informed consent given this was the registry EHR study with anonymized data.

### Study population

Consecutive patients with AF *admitted* to the PLA General Hospital, Beijing, China between January 1, 1995 to May 30, 2015 were included. Inclusion criteria were adult AF population age over 18 years, with documented evidence of AF on ECG or 24 h Holter recording. Exclusion criteria included patients age <18 years and outpatients.

Thus, a total of 7479 inpatients with AF were initially selected, and after excluding 11 inpatients aged under 18 years, we identified 7468 adult patients with AF. Of these, 2555 patients with readmission(s) were excluded, leaving 4824 adult patients with AF for the final analysis [Suppl. Figure w1].

### Bleeding Risk Scores

The mOBRI, HEMORR2HAGES, Shireman, HAS-BLED, ATRIA, and ORBIT scores were calculated for this cohort.

#### OBRI score

Age ≥65 years, previous stroke, gastro-intestinal bleed, ≥1 of the following comorbidities [recent MI, haematocrit <30%, creatinine >1.5 mg/dl, or diabetes mellitus] with 1 point for presence of each risk factor and 0 if absent. Low risk: 0, intermediate risk: 1–2, high risk: ≥3.

#### HEMORR_2_HAGES score

Hepatic or renal disease, Ethanol abuse, Malignancy, Older (aged >75), Reduced platelet count, Re-bleeding risk, uncontrolled Hypertension, Anaemia, Genetic factors (CYP 2C9 single nucleotide polymorphisms), Excessive fall risk, previous Stroke/TIA, 1 point for each risk factor present, & 2 points for previous bleed. Low risk: 0–1, intermediate risk: 2–3, high risk: ≥4. Genetic factors were not routinely measured, so this criterion scored 0, as per previous studies.

#### Shireman score

[0.49 × age ≥70] + [0.32 × female gender] + [0.58 × remote bleed] + [0.62 × recent bleed] + [0.71 × alcohol/drug abuse] + [0.27 × diabetes] + [0.86 × anaemia] + [0.32 × antiplatelet] with 1 point for the presence of each condition and 0 if absent. Low risk: ≤1.07, intermediate risk: >1.07 to <2.19, high risk: ≥2.19.

#### HAS-BLED score

Hypertension (uncontrolled systolic BP >160 mm Hg), Abnormal renal/liver function, Stroke, Bleeding history, Labile INR, Elderly (age >65 years), Drugs (antiplatelets/NSAIDS)/concomitant alcohol (≥8 units/week), with 1 point for the presence of each risk factor. Low risk: 0–1, intermediate risk: 2, high risk: ≥3. For the labile INR criterion, we recorded ‘poor anticoagulation control’ as assessed by the responsible physician or INR <2.0 at presentation.

#### ATRIA score

Anaemia, severe renal disease (estimated glomerular filtration rate, eGFR <30 ml/min or dialysis-dependent), age ≥75 years, previous bleed, hypertension, with 1 point each for presence of previous bleed or hypertension, 2 points for age ≥75, and 3 points each for presence of anaemia and renal disease. Low risk: 0 to 3, intermediate risk: 4, high risk: 5 to 10.

#### ORBIT score

1 point each for age >74, insufficient kidney function (eGRF <60 ml/min/1.73 m^2^) and treatment with any antiplatelet, while 2 points were assigned to a positive clinical history for bleeding and the presence of anaemia or abnormal hemoglobin (<13 mg/dL for males and <12 mg/dL for females). Low risk: 0 to 2, intermediate risk: 3, high risk: ≥4.

### Evaluation of bleeding events, co-morbidities and risk factors

Major bleeding was defined according to International Society on Thrombosis and Haemostasis (ISTH) criteria, as follows: (i) fatal bleeding; and/or (ii) symptomatic bleeding in a critical area or organ (intracranial, intraspinal, intraocular, retroperitoneal, intra-articular or pericardial, or intramuscular with compartment syndrome); and/or (iii) bleeding causing a fall in hemoglobin level of 20 g/L or more, or leading to transfusion of two or more units of whole blood or red cells^24^. ICH (intracerebral and subarachnoid haemorrhage) was defined as “a focal or global neurologic deficit of sudden onset, developing clinical symptoms and/or signs, loss of cerebral function, with symptoms lasting more than 24 hours or leading to death”, diagnosed clinically by a neurologist and ICH confirmed by computed tomography (CT) scanning or magnetic resonance imaging (MRI).

Information on ICH, and co-morbidities were based on ICD-9 and ICD-10 codes. ICH cases were identified by ICD-9 or ICD-10 codes 430,431,432; I60.x, I61.x. Other major bleeding [ICD-10 codes: I85.0, I98.3 K25–28 (subcodes 0–2 and 4–6 only); K62.5, K92.2, D62.9], heart failure (ICD-9 codes:428; ICD-10 codes: I42, I50, I110, J819), dilated cardiomyopathy (ICD-9 codes:425.4; ICD-10 codes: I42.0), diabetes (ICD-9 codes:249–250; ICD-10 codes: E10-E14), hypertension (ICD-9 codes:401–405; ICD-10 codes: I10-I15), coronary artery disease (ICD-9 codes:410–414; ICD-10 codes: I20-I25), myocardial infarction (ICD-9 codes:410; ICD-10 codes: I21, I22), peripheral vascular disease (ICD-9 codes:440.2; ICD-10 codes: I65, I70–74), chronic obstructive pulmonary disease (ICD-9 codes:490–496; ICD-10 codes: J42, J44.0–9), hyperlipidaemia (ICD-9 codes:272.4; ICD-10 codes: E78.0–3, E78.5), renal dysfunction (ICD-9 codes:585, 586; ICD-10 codes: M1A.3), hyperthyroidism (ICD-9 codes:242; ICD-10 codes: E05), hypothyroidism (ICD-9 codes:244; ICD-10 codes: E03). ICD-9, ICD-10 codes defined cardiovascular disease and other co-morbidities are shown in Suppl. Table w1.

For this analysis, AF and comorbidities were identified based on ICD9/10 codes using the electronic medical records database (Table w1). The detail definitions of various co-morbidities are summarized in Suppl. Table w2. Bleeding events and bleeding scores were calculated using ICD9/10 codes (eg. ICH, gastrointestinal bleeding, etc.), laboratory tests (eg. a decrease in hemoglobin level of 20 g/L or more for major bleeding; hemoglobin <13 mg/dL for males and <12 mg/dL for females for bleeding risk scores etc.), and medical records (eg. transfusion of two or more units of whole blood or red cells, etc.). Major bleeding events were defined by standard International Society for Thrombosis and Haemostasis (ISTH) criteria using information above.

### Bleeding risk scores and major bleeding on admission and in-hospital period

Major bleeding and ICH of AF patients on admission and during the in-hospital period were identified between 1995–2015. Bleeding risk of AF patients was evaluated by the available bleeding risk scores on admission, and the association between the bleeding risk scores and major bleeding/ICH on admission and during the in-hospital period was analyzed.

### Statistical analysis

Continuous variables were tested for normality by the Kolmogorov-Smirnov test. Those with a normal distribution are presented as a mean (standard deviation, SD), and data with a non-normal distribution are presented as median (interquartile range, IQR). A multivariate analysis (binary logistic model regression) was used to analyze the association of clinical risk factors and the occurrence of major bleeding events in this cohort.

The distribution of bleeding risk scores (mOBRI, HEMORR_2_HAGES, Shireman, HAS-BLED, ATRIA, and ORBIT) as low, intermediate, and high categories in the 4824 AF patients, was first described, as were major bleeding events by risk category. The ability of the bleeding scores to predict major bleeding and ICH were assessed by Receptor Operating Characteristic Curve (ROC) analyses, and expressed by C-indexes (95% confidence intervals (CI)). As an age-stratified analysis, the predictive ability of the bleeding scores were also explored in subjects age ≥65 years and <65 years, respectively. To assess impact of time trends, a sensitivity analysis of the predictive value of bleeding scores was performed amongst AF patients from 1995 to 2005, and from 2005 to 2015.

To compare the diagnostic accuracy of the available risk scores, the differences of areas under the curve (AUC, C-statistic) were tested for significance by the DeLong equality test. We also used the net reclassification improvement (NRI)^25^ to further verify the predictive and discriminatory ability for major bleeding and ICH of the six bleeding risk scores.

A two-sided P-value < 0.05 was considered as statistically significant. The 95% confidential intervals (CIs) were calculated and statistical analysis was performed using IBM SPSS Statistics, version 22.0 (SPSS Inc) and MedCalc 12.6.1.0 (MedCalc Software).

## Results

A total of 4824 adult patients with AF (mean age 67 years; 34.9% female) were studied during the 20-year observational period. The median (interquartile) in-hospital stay was 10 (7–16) days (Table 1). Of these, 481 (10%) were on OAC, with 450 patients taking warfarin and 31 taking non-vitamin K antagonist coagulant (NOAC) (Table 1). Of the patients on warfarin, 96% had INR on admission of <2. Hypertension was the most prevalent co-morbidity, followed by CAD. The mean and median values for the bleeding risk scores in 4824 Chinese AF patients are shown in Suppl. Table w3.

The rate of major bleeding (95% CI) was 1.14% (0.88–1.48%), including 0.52% (0.35–0.76%) with ICH and 0.62% (0.44–0.89%) with extracranial bleeding (Suppl. Table w4).

### Multivariate analyses

On multivariate analysis, prior bleeds, vascular disease (carotid atherosclerosis, peripheral vascular disease, vascular amyloidosis, vascular dementia), anaemia, prior stroke, and liver dysfunction were the independent risk factors of major bleeding events (all p < 0.05) (Table 2).

### Bleeding scores and related bleeding events

The proportions of patients in relation to low, intermediate and high risk strata associated with the mOBRI, Shireman, HEMORR_2_HAGES, HAS-BLED, ATRIA, and ORBIT scores are shown in Suppl. Figure 2.

**Figure 1:**
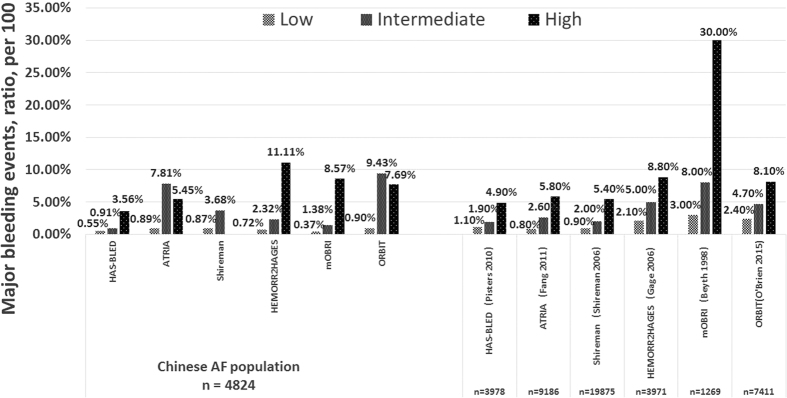
Major bleeding events by risk category in 4824 AF patients with HAS-BLED, ATRIA, Shireman, HEMORR2HAGES, mOBRI, and ORBIT scores, in related to the derivate western population.

Major bleeding and ICH rates (bleeds per 100, 95% CI) categorized by risk category (low, intermediate and high risk) associated with bleeding risk schemes were showed in Table w5, in comparison to published rates from the derived western population (Fig. 1).

Of the cohort, 60.3% were low risk, 22.8% intermediate risk, and 16.9% high risk, when stratified by HAS-BLED score. ORBIT categorized 97% as ‘low risk’ and 0.8% as ‘high risk’, whilst for mORBI, the figures were 33.9% and 1.5%, respectively. The rates (95% CI) of major bleeding in patients at low, intermediate, and high risk with HAS-BLED score were 0.55 (0.34–0.89), 0.91 (0.49–1.66), and 3.56 (2.49–5.06), respectively (Table w5). The increased trend of major bleeding event with HAS-BLED risk strata in this cohort was similar to that in the EurHeart survey with Pisters *et al*.^14^, but the stepwise increase in bleeding with risk categories was less evident with other scores and different from their respective derivation (usually Western) cohorts (Fig. 1).

### Predictive ability of risk scores in the Chinese AF cohort

ROC analyses showed that HAS-BLED had the best predictive ability of bleeding risk, with C statistics (95% CI) of 0.72 (0.65–0.79) for major bleeding and 0.83 (0.75–0.91) for ICH (all p < 0.001) (Table 3). The significant predictive ability of HAS-BLED was also seen in the elderly (age ≥65) (major bleeding and ICH: 0.71 (0.65–0.77) and 0.80 (0.73–0.88), respectively, all p < 0.001) (see Suppl. Table w6).

In a sensitivity analysis, we also examined the predictive ability of different bleeding risk scores, by 2 time periods: 1995 to 2005, and 2005 to 2015. Findings from these 2 time periods were consistent with the main findings in the overall cohort (see Suppl. Table w7).

**Figure 2:**
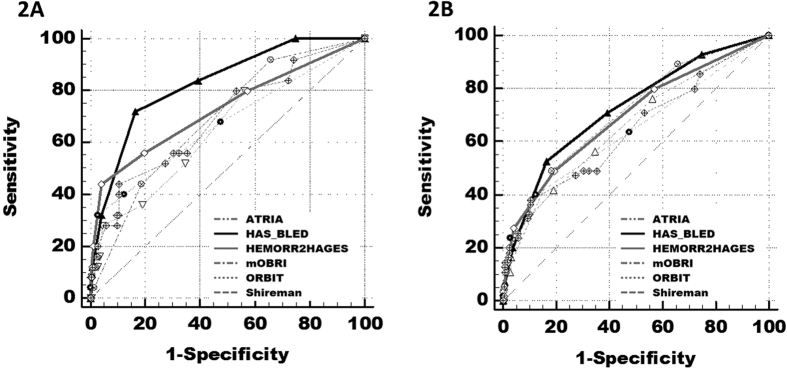
Comparison of ROC curves of HAS-BLED, ATRIA, Shireman, HEMORR2HAGES, mOBRI, and ORBIT scores for intracranial haemorrhage and major bleeding events with Delong test. 2A Intracranial haemorrahge. 2B Major bleeding events.

The HAS-BLED score was superior to other bleeding scores in predicting bleeding risks, with the difference between AUC areas of 0.10–0.17 for ICH (Delong test, all P < 0.05) and of 0.02–0.08 for major bleeding events (all P < 0.05, apart from mOBRI, HEMORR_2_HAGES) (Table 4Fig. 2A,B). Compared to other bleeding risk schemes, the use of the HAS-BLED score would result in the NRI of 29.5–67.3% in predicting ICH and of 17.1–65.5% in predicting major bleeding events (all p < 0.05) (Table 4).

## Discussion

In the present study, our principal findings were the HAS-BLED score having the best predictive ability for major bleeding and ICH in an Asian/Chinese AF population, compared to the mOBRI, Shireman, HEMORR_2_HAGES, ATRIA and ORBIT scores. Second, prior bleeding, vascular disease (carotid atherosclerosis, peripheral vascular disease, vascular amyloidosis, vascular dementia), anemia, prior stroke, and liver dysfunction were independent factors associated with major bleeding risk in this population.

The superior predictive ability of HAS-BLED for ICH was consistent in the young and elderly population. The predictive ability of HAS-BLED for major bleeding events was maintained even in the elderly AF population age >65 years. The elderly is particularly prone to an increased risk of bleeding and ICH^26^, and our analysis in a large Asian/Chinese population clearly shows that the HAS-BLED score can be used to assess risk in these patients. However, a high HAS-BLED score is not an excuse to withhold OAC but to ‘flag up’ the patients potentially at risk of bleeding for more careful review and follow-up, and to address the potentially reversible bleeding risk factors.

Various publications have consistently shown a low rate of OAC use in the Chinese AF population^4^,^27^. The fear of bleeding may contribute to the low OAC use, although quality of anticoagulation control is also important given the close (inverse) relationship of time in therapeutic INR range (TTR) to stroke, mortality and bleeding^28^,^29^. In this AF cohort we also see a low rate of OAC use (10%), and prior bleeds, vascular disease, anemia, prior stroke, and liver dysfunction independently predict the risk for major bleeding events. Of these, vascular disease (vascular amyloidosis, etc.) may be an important driver of major bleeding events (over six-fold increased risk in this cohort, as does a predisposition to bleeding event (e.g. Prior bleeds, anemia).

AF patients show a propensity to a higher risk of bleeding, even in the absence of antithrombotic therapy^30^. Our previous studies from Chinese hospital and community longitudinal AF cohorts demonstrate that the annual major bleeding episodes was 1–1.5% and the annual ICH rate was 0.4%, with the rate of warfarin use being 6–14%^3^,^8^. In a Taiwanese cohort without antithrombotic therapy, the incidence of major bleeding events and ICH was 4.5 per 100 person-years and 0.87% per 100 person-years, respectively^25^. These rates may be higher than reported rates from clinical trials or non-Asian populations. For example, reported rates of major bleeding among western populations with AF taking oral VKA vary from 1.3% to 7.4%, and ICH rates, from 0.3–2.5%^9^.

Asians are especially prone to ICH^4^,^31^,^32^, with more than two-fold excess risk compared to Caucasians and four-fold increased risk when receiving the warfarin^6^,^7^. In four landmark randomized control trials with NOACs, the Asian population with OACs had a significant predisposition to ICH with 2.11% (95% CI, 1.77–2.50%) in Asian population compared to 0.97% (0.89–1.05%) in non-Asian population (p < 0.001)^33^.

In comparison to mOBRI, Shireman, HEMORR_2_HAGES, ATRIA, and ORBIT, we show that the HAS-BLED score demonstrates that the best predictive ability for major bleeding events, especially ICH, in this large Asian population with AF. As far as we are aware, this is the largest Asian/Chinese cohort to compare the published bleeding risk scores, which also includes the newer ATRIA and ORBIT scores. Of note, the HAS-BLED score improved the predictive ability for ICH by 37.5% compared to ORBIT and 32.4% compared to ATRIA, and improved the predictive ability for major bleeding events of these scores by 25.3% and 17.1%, respectively. Good discrimination in predicting ICH using HAS-BLED has also been shown in a non-AF Chinese population^34^, consistent with multiple collective co-morbidities contributing to the bleeding risk. Of note, the predictive ability for major bleeding events and ICH of HAS-BLED was consistent in the elderly population age over 65 years.

### Limitations

The main limitation of the study was its dependence on our hospital electronic medical records database. However, our detailed in-hospital records, including the patient’s medical history, therapeutic procedure, laboratory data (based on Laboratory Information System, LIS), and imaging data (based on Picture Archiving and Communications System, PACS), allowed use to accurately collect the risk factors and co-morbidities, which were components of the different risk scores. In calculating HEMORR2HAGES risk scores, the genetic factor (CYP 2C9 single nucleotide polymorphisms) criterion was not available, which may arguably reduce the precision of the original risk score. In our cohort, there were only 9% patients on warfarin, most of those who had suboptimal INR on admission (i.e. <2.) reflecting the generally poor quality of anticoagulation control in China. Also, there were 336 (6.97%) patients with rheumatic heart disease in this cohort, but excluding these patients from our analysis did not change our conclusions [data not shown].

Moreover, the relative small number of ICHs (n = 25) in this cohort could influence statistical power in terms of assessing the predictive value of different bleeding risk scores for ICH. Nonetheless, our findings were consistent with our analysis of the predictive value of bleeding scores for major bleeding.

Finally, this cohort included a typical ‘real world’ Chinese AF population on OAC, ASA or no antithrombotic drugs. In prior ‘real-world’ studies, the Chinese AF population has a low rate of OAC use, with more antiplatelet drugs or no antithrombotic therapy use (eg. warfarin use: 0.5–4% in community-based data, 6.6–20% from hospital-based data; aspirin use: 33% in community-based data, 40–60% in hospital-based data, etc.) often due to the fear of bleeding^3^,^8^,^35^,^36^. The HAS-BLED score has previously been validated in OAC, ASA or no antithrombotic drug use, and in AF and non-AF populations.

## Conclusions

The HAS-BLED score had the best predictive ability for major bleeding and ICH in an Asian/Chinese AF population. Prior bleeding, vascular disease, anemia, prior stroke, and liver dysfunction were associated with major bleeding risk in this population.

## Additional Information

**How to cite this article**: Guo, Y.-t. *et al*. Assessing bleeding risk in 4824 Asian patients with atrial fibrillation: The Beijing PLA Hospital Atrial Fibrillation Project. *Sci. Rep.* **6**, 31755; doi: 10.1038/srep31755 (2016).

## Supplementary Material

Supplementary Information

## Figures and Tables

**Table 1:** Clinical and demographic characteristics of 4824 patients with Atrial Fibrillation.

	AF (n = 4824)	
Age, mean(SD)	67	(13)
Female, n(%)	1685	(34.93%)
Medical history		
Hypertension, n(%)	1886	(39.10%)
CAD, n(%)	1403	(29.08%)
Diabetic, n(%)	667	(13.83%)
RHD, n(%)	336	(6.97%)
Renal dysfunction, n(%)	216	(4.50%)
HF, n(%)	151	(3.13%)
Dilated cardiomyopathy, n(%)	108	(2.24%)
COPD, n(%)	103	(2.14%)
Anemia, n(%)	77	(1.60%)
Vascular disease, n(%)	58	(1.20%)
Liver dysfunction, n(%)	50	(1.04%)
Hypertrophy cardiomyopathy, n(%)	32	(0.66%)
CHADS_2_ scores, median(quartile)	1	(0–2)
CHA_2_DS_2_-VASc scores, median(quartile)	2	(1–3)
Drugs		
ACEI/ARB	707	(14.66%)
Beta blocker	1072	(22.22%)
Statin	866	(17.95%)
Other lipid control drug	50	(1.04%)
Digoxin	17	(0.35%)
Amiodarone	349	(7.23%)
Propafenone	166	(3.44%)
Diuretic	532	(11.03%)
CCB	412	(8.54%)
Nitrates	88	(1.82%)
Insulin	66	(1.37%)
Sulfonylureas	44	(0.91%)
Biguanides	131	(2.72%)
Proton pump inhibitors, PPI	251	(5.20%)
Nicorandil	31	(0.64%)
Aspirin	1022	(21.19%)
Clopidogrel	345	(7.15%)
Ticagrelor	13	(0.27%)
Prasugrel	1	(0.02%)
Warfarin	450	(9.33%)
Dabigatran	21	(0.44%)
Rivaroxaban	10	(0.21%)

*CAD: coronary artery disease. RHD: rheumatic heart disease. HF: heart failure. COPD: chronic obstructive pulmonary disease. Vascular disease: carotid atherosclerosis, peripheral vascular disease, vascular amyloidosis, vascular dementia. ACEI/ARB: angiotensin-converting-enzyme inhibitor, angiotensin receptor blockers. CCB: calcium channel blockers.

**Table 2:** Multivariate analysis of major bleeding in 4824 patients with AF.

	OR	95%CI		P
		Lower limit	Higher limit	
**Prior bleeds**	13.82	5.52	34.61	**<0.001**
**Vascular disease**	6.24	2.24	17.39	**<0.001**
**Anemia**	6.19	2.21	17.34	**0.001**
**Prior stroke**	5.00	2.62	9.53	**<0.001**
**Liver dysfunction**	3.91	1.03	14.90	**0.045**
Hypertension	1.70	0.89	3.24	0.106
Heart failure	1.27	0.35	4.57	0.714
Age	1.00	0.98	1.03	0.840
Diabetes	0.87	0.43	1.77	0.699
Female	0.85	0.47	1.55	0.604
Renal dysfunction	0.84	0.30	2.34	0.734
Antiplatelet	0.72	0.37	1.42	0.344

*OR: odds ratio. Vascular disease: carotid atherosclerosis, peripheral vascular disease, vascular amyloidosis, vascular dementia.

**Table 3:** Comparison of predictive ability of major bleeding with different bleeding risk scores in 4824 Chinese patients with AF.

Major bleeding (n = 55)	C statistic	95% CI	p
HAS-BLED	0.72	0.65–0.79	<0.001
mOBRI	0.70	0.63–0.77	<0.001
HEMORR_2_HAGES	0.69	0.62–0.77	<0.001
ATRIA	0.66	0.58–0.74	<0.001
ORBIT	0.64	0.56–0.73	<0.001
Shireman	0.64	0.55–0.72	<0.001
**Intracranial haemorrhage (n = 25)**			
HAS-BLED	0.83	0.75–0.91	<0.001
HEMORR_2_HAGES	0.73	0.61–0.85	<0.001
Shireman	0.69	0.58–0.80	<0.001
mOBRI	0.69	0.59–0.78	<0.001
ORBIT	0.67	0.54–0.79	<0.001
ATRIA	0.66	0.54–0.76	<0.001

*CI: confidential interval.

**Table 4:** Comparison of predicting intracranial haemorrhage and major bleeding of HAS-BLED and other bleeding risk scores.

	ROC curves analysis			NRI analysis	
**Intracranial haemorrhage**	Difference between areas (95%CI)	Z score	p	NRI (95%CI)	p
HAS-BLED vs. ATRIA	0.17 (0.08–0.26)	3.640	<0.001	0.324 (0.321–0.327)	<0.001
HAS-BLED vs. ORBIT	0.16 (0.07–0.25)	3.576	0.003	0.375 (0.373–0.378)	<0.001
HAS-BLED vs. Shireman	0.14 (0.05–0.22)	3.244	0.001	0.435 (0.432–0.438)	<0.001
HAS-BLED vs. mOBRI	0.14 (0.06–0.22)	3.274	0.001	0.673 (0.670–0.676)	<0.001
HAS-BLED vs. HEMORR_2_HAGES	0.10 (0.01–0.19)	2.011	0.044	0.295 (0.292–0.298)	<0.001
**Major bleeding**					
HAS-BLED vs. Shireman	0.08 (0.02–0.15)	2.560	0.010	0.290 (0.288–0.292)	<0.001
HAS-BLED vs. ORBIT	0.08 (0.01–0.14)	2.441	0.015	0.253 (0.251–0.254)	<0.001
HAS-BLED vs. ATRIA	0.06 (0.00–0.12)	2.024	0.043	0.171 (0.169–0.172)	<0.05
HAS-BLED vs. HEMORR_2_HAGES	0.03 (−0.02−0.08)	1.130	0.258	0.242 (0.240–0.243)	<0.001
HAS-BLED vs. mOBRI	0.02 (−0.04−0.09)	0.679	0.497	0.655 (0.653–0.656)	<0.001

*ROC: receiver operating characteristic. NRI: net reclassification improvement.
