# Supplementary material for: Assessing bleeding risk in 4824 Asian patients with atrial fibrillation: The Beijing PLA Hospital Atrial Fibrillation Project
Source: Sci Rep. 2016 Aug 25;6:31755. doi: 10.1038/srep31755 (PMC4997334; doi:10.1038/srep31755)
Supplement: Supplementary Information [file srep31755-s1.pdf]

SUPPLEMENTARY MATERIAL: Assessing bleeding risk in 4824 Asian patients with atrial fibrillation: The Beijing PLA Hospital Atrial Fibrillation Project

Yu-tao Guo [1], Ye Zhang[1], Xiang-min Shi[1], Zhao-liang Shan[1], Chun-jiang Wang[2], Yu-tang Wang[1], Yun-dai Chen [1,*] & Gregory Y H Lip[3,*]

Table w1 Comorbidity ICD codes

Comorbidity	ICD-9 codes	ICD-10 codes
Rheumatic heart disease	393–398	I05,I06,I07,I09.9
Heart failure	428	I42, I50, I110,J819
Dilated cardiomyopathy	425.4	I42.0
Diabetes	249–250	E10-E14
Hypertension	401–405	I10-I15
Coronary artery disease	410-414	I20-I25
Myocardial infarction	410	I21, I22
Peripheral vascular disease	440.2	I65, I70-74
Ischemic stroke	436	I63
Intracranial haemorrhage	430,431,432	I60.x,I61.x
Hemoptysis	786.3	R04.2
Gastrointestinal bleeding	R04.201, J47.X02	K92.208,K92.204,K92.207, K27.404
Chronic obstructive pulmonary disease	490–496	J42,J44.0-9
Hyperlipidemia	272.4	E78.0-3,E78.5
Renal dysfunction	585,586	M1A.3
Hyperthyroidism	242	E05
Hypothyroidism	244	E03
Atrial fibrillation	427.31	I48

Table w2 Definitions of comorbidities

Comorbidity	Definition
Hypertension	A resting blood pressure ≥ 140mmHg systolic and/or ≥90 mmHg diastolic on at least 2 occasions or current antihypertensive drug treatment
Coronary artery disease	Prior myocardial infarction, angina pectoris, percutaneous coronary intervention or coronary artery bypass surgery
Heart failure	The presence of signs and symptoms of either right (elevated central venous pressure, hepatomegaly, dependent edema) or left ventricular failure (exertional dyspnea, cough, fatigue, orthopnea, paroxysmal nocturnal dyspnea, cardiac enlargement, rales, gallop rhythm, pulmonary venous congestion) or both, confirmed by non-invasive or invasive measurements demonstrating objective evidence of cardiac dysfunction
Peripheral vascular disease	Intermittent claudication, previous surgery or percutaneous intervention on the abdominal aorta or the lower extremity vessels, abdominal or thoracic surgery, arterial and venous thrombosis
Diabetes	Fasting plasma glucose concentration ≥ 7.0 mmol/l, or two hour plasma glucose concentration ⩾ 11.1 mmol/l after 75g anhydrous glucose in an oral glucose tolerance test
Rheumatic heart disease	Damaged heart valves that can be a narrowing or leakage, particularly mitral valve (mitral valve stenosis) as a result of residual symptoms of rheumatic fever
Dilated cardiomyopathy	The heart becomes weakened and enlarged, and it cannot pump blood efficiently.
Renal dysfunction	The presence of chronic dialysis, renal transplantation, or an estimated glomerular filtration rate (eGFR) <60 mL/min

	per 1.73 m^2
Liver dysfunction	The presence of liver disease with serum levels of either ALT (SGPT), AST (SGOT), or alkaline phosphatase above 3 x upper limit of normal (ULN)
Hyperlipidemia	An elevation of lipids or lipoproteins in the blood, e.g. fasting triglyceride level >200 mg/dL, LDL-L>160mg/dL , and non-HDL-C>190mg/dl
Hyperthyroidism	High levels of thyroxine and low or nonexistent amounts of thyroid stimulating hormone (TSH)
Hypothyroidism	Low thyroxine and high TSH levels
Chronic obstructive pulmonary disease	A nonreversible lung disease that is a combination of emphysema and chronic bronchitis
Ischemic stroke	A focal neurologic deficit of sudden onset diagnosed clinically by a neurologist based on CT or MRI scanning

Table w3 Mean and median scores with the available bleeding risk stratification schemes in 4824 Chinese AF patients

	median(IQR)	mean (SD)
mOBRI	1(0-1)	0.86(0.74)
HEMORR$_2$HAGES	1(0-1)	0.83(0.90)
Shireman	0.49(0.00-0.81)	0.47(0.38)
HAS-BLED	1(1-2)	1.37(1.14)
ATRIA	1(0-2)	0.83(0.90)
ORBIT	0(0-1)	0.65(0.82)

* IQR: interquartile. SD： standard deviation

Table w4 Major bleeding rates in 4824 AF patients

Major bleeding	n	Rate (95%CI)
Intracranial haemorrhage	25	0.52% (0.35%-0.76%)
Extracranial bleeding	30	0.62% (0.44%-0.89%)
GI bleeding	17	0.35% (0.22%-0.56%)
Respiratory bleeding	3	0.06% (0.02%-0.18%)
Hematoma	2	0.04% (0.01%-0.15%)
Other major bleeding	8	0.16% (0.08%-0.33%)
**Total**	**55**	**1.14%（0.88%-1.48%）**

*Intracranial haemorrhage: including haemorrhagic stroke, subarachnoid haemorrhage, subdural haematoma, and epidural haemorrhage. GI bleeding: gastrointestinal bleeding, including upper gastrointestinal bleeding, lower gastrointestinal bleeding, and internal hemorrhoids with major bleeding. Hematoma: including retroperitoneal hematoma, inguinal hematoma, hematoma of abdominal wall, limber hematoma. Other major bleeding: including urinary bleeding, subcutaneous hemorrhage resulting in anemia, and fundus hemorrhage.

Table w5 Major bleeding events (bleeds per 100 person-years, 95% CI) classified by risk category

**Major bleeding (n=55)**	Low	Intermediate	High
HAS-BLED	0.55(0.34-0.89)	0.91(0.49-1.66)	3.56(2.49-5.06)
ATRIA	0.89(0.66-1.21)	7.81(3.38-17.02)	5.45(2.90-10.04)
Shireman	0.87(0.64-1.20)	3.68(2.31-5.81)	0(0)
HEMORR$_2$HAGES	0.72(0.50-1.04)	2.32(1.52-3.53)	11.11(5.19-22.19)
mOBRI	0.37(0.17-0.80)	1.38(1.03-1.85)	8.57(3.99-17.47)
ORBIT	0.90(0.66-1.21)	9.43(5.21-16.50)	7.69(2.65-20.32)
**Intracranial haemorrhage (n=25)**	Low	Intermediate	High
HAS-BLED	0.14（0.05-0.35）	0.27（0.09-0.80）	2.21（1.40-3.46）
ATRIA	0.391（0.25-0.62）	4.69（1.61-12.9）	2.42（0.95-6.07）
Shireman	0.41（0.26-0.64）	1.51（0.74-3.09）	0（0）
HEMORR$_2$HAGES	0.28（0.16-0.51）	0.10（0.52-1.88）	9.26（4.02-19.91）
mOBRI	0.12（0.03-0.45）	0.71（0.47-1.07）	1.43（0.25-7.66）
ORBIT	0.36(0.23-0.58)	5.66(2.62-11.80)	5.13(1.42-16.89)

* HAS-BLED: low 0-1, Intermediate 2, High ≥3; ATRIA: low 0-3, Intermediate 4, High 5-10; Shireman: low ≤ 1.07, Intermediate >1.07 to <2.19, High ≥ 2.19; HEMORR2HAGES: low 0-1, Intermediate 2-3, High ≥4; mOBRI: low 0, Intermediate 1-2, High ≥3. ORBIT: low 0-2, Intermediate 3, High: ≥4

Table w6 Predictive ability of major bleeding with different bleeding risk scores, in relation to age strata

Age≥65 (n=2876)				Age<65 (n=1948)			
Major bleeding (n=41)	C statistic	95% CI	p	Major bleeding events (n=14)	C statistic	95% CI	p
HAS-BLED	0.71	0.65-0.77	<0.001	OBRI	0.68	0.53-0.83	0.014
HEMORR2HAGES	0.67	0.59-0.74	<0.001	HEMORR2HAGES	0.66	0.51-0.81	0.027
OBRI	0.64	0.56-0.71	<0.001	HAS-BLED	0.65	0.50-0.79	0.044
ATRIA	0.63	0.55-0.71	<0.001	ATRIA	0.61	0.45-0.77	0.134
ORBIT	0.61	0.53-0.69	0.002	Shireman	0.58	0.42-0.74	0.276
Shireman	0.57	0.48-0.65	0.077	ORBIT	0.55	0.39-0.71	0.465
Intracranial haemorrhage (n=20)	C statistic	95% CI	p	Intracranial haemorrhage (n=5)	C statistic	95% CI	p
HAS-BLED	0.80	0.73-0.88	<0.001	HAS-BLED	0.76	0.57-0.96	0.027
HEMORR2HAGES	0.69	0.58-0.82	<0.001	HEMORR2HAGES	0.73	0.49-0.97	0.048
ORBIT	0.63	0.51-0.74	0.020	ATRIA	0.73	0.4900.97	0.051
Shireman	0.60	0.48-0.72	0.071	OBRI	0.67	0.43-0.92	0.143
ATRIA	0.60	0.48-0.73	0.061	ORBIT	0.60	0.33-0.87	0.384
OBRI	0.59	0.48-0.70	0.091	Shireman	0.55	0.27-0.82	0.672

***** 95% CI: confidential interval.

Table w7 Predictive ability of major bleeding with different bleeding risk scores, in relation to time period

**1995 year to 2005 year** (n=1168)				**2005 year to 2015 year** (n=3656)			
Major bleeding (n=11)	C statistic	95% CI	p	Major bleeding (n=44)	C statistic	95% CI	p
HAS-BLED	0.72	0.58-0.87	0.011	HAS-BLED	0.73	0.65-0.82	<0.001
mOBRI	0.69	0.59-0.80	0.026	mOBRI	0.70	0.61-0.78	<0.001
HEMORR2HAGES	0.72	0.64-0.80	0.012	HEMORR2HAGES	0.69	0.60-0.79	<0.001
ATRIA	0.66	0.55-0.77	0.064	ATRIA	0.66	0.65-0.67	<0.001
ORBIT	0.52	0.35-0.68	0.860	ORBIT	0.68	0.59-0.78	<0.001
Shireman	0.51	0.38-0.65	0.888	Shireman	0.68	0.58-0.77	<0.001
Intracranial haemorrhage (n=5)	C statistic	95% CI	p	Intracranial haemorrhage (n=20)	C statistic	95% CI	p
HAS-BLED	0.77	0.64-0.90	0.036	HAS-BLED	0.86	0.79-0.94	<0.001
HEMORR2HAGES	0.70	0.53-0.88	0.119	HEMORR2HAGES	0.72	0.58-0.87	0.001
Shireman	0.47	0.25-0.69	0.804	Shireman	0.75	0.64-0.86	<0.001
mOBRI	0.59	0.37-0.81	0.477	mOBRI	0.71	0.61-0.82	0.001
ORBIT	0.44	0.21-0.67	0.626	ORBIT	0.73	0.60-0.86	<0.001
ATRIA	0.68	0.56-0.80	0.166	ATRIA	0.65	0.52-0.79	0.017

***** 95% CI: confidential interval.

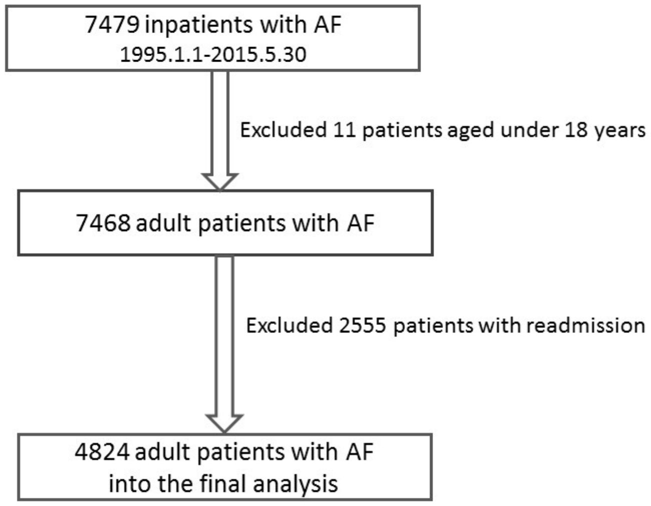

Figure w 1 Patient flow chart

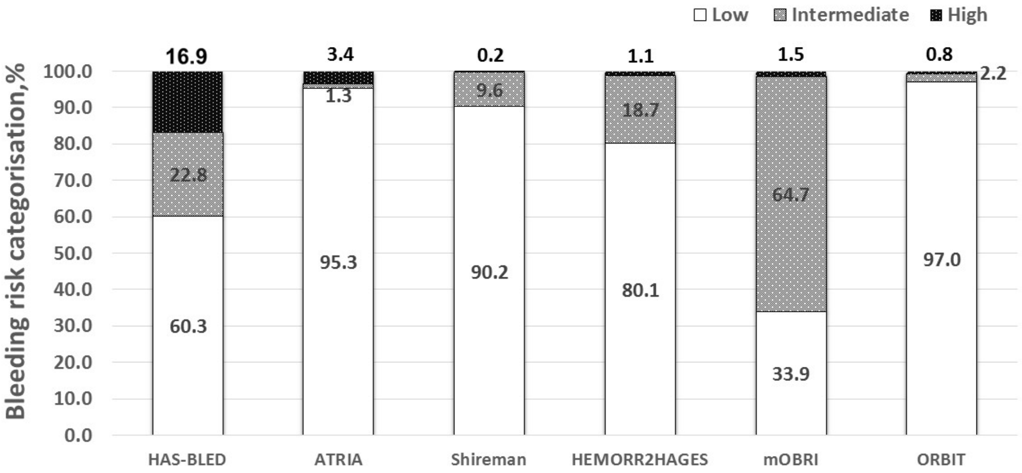

Figure w 2 Bleeding risk categorisation with HAS-BLED, ATRIA, Shireman, HEMORR2HAGES, mOBRI, and ORBIT scores in 4824 AF patients
